# Macrophages of genetically characterized familial hypercholesterolaemia patients show up‐regulation of LDL‐receptor‐related proteins

**DOI:** 10.1111/jcmm.12993

**Published:** 2016-09-29

**Authors:** Rafael Escate, Teresa Padro, Maria Borrell‐Pages, Rosa Suades, Rosa Aledo, Pedro Mata, Lina Badimon

**Affiliations:** ^1^ Cardiovascular Research Center (CSIC‐ICCC)IIB‐Sant PauBarcelonaSpain; ^2^ Cardiovascular Research ChairUABBarcelonaSpain; ^3^ Foundation Jimenez DiazMadridSpain

**Keywords:** atherosclerosis, macrophages, familial hypercholesterolaemia, LRPs, CD163

## Abstract

Familial hypercholesterolaemia (FH) is a major risk for premature coronary heart disease due to severe long‐life exposure to high LDL levels. Accumulation of LDL in the vascular wall triggers atherosclerosis with activation of the innate immunity system. Here, we have investigated (i) gene expression of *LDLR* and LRPs in peripheral blood cells (PBLs) and in differentiated macrophages of young FH‐patients; and (ii) whether macrophage from FH patients have a differential response when exposed to high levels of atherogenic LDL. PBLs in young heterozygous genetically characterized FH patients have higher expression of *LRP5* and *LRP6* than age‐matched healthy controls or patients with secondary hypercholesterolaemia. *LRP1* levels were similar among groups. In monocyte‐derived macrophages (MACs), *LRP5* and *LRP1* transcript levels did not differ between FHs and controls in resting conditions, but when exposed to agLDL, FH‐MAC showed a highly significant up‐regulation of *LRP5*, while *LRP1* was unaffected. PBL and MAC cells from FH patients had significantly lower *LDLR* expression than control cells, independently of the lipid‐lowering therapy. Furthermore, exposure of FH‐MAC to agLDL resulted in a reduced expression of *CD163*, scavenger receptor with anti‐inflammatory and atheroprotective properties. In summary, our results show for first time that LRPs, active lipid‐internalizing receptors, are up‐regulated in innate immunity cells of young FH patients that have functional LDLR mutations. Additionally, their reduced *CD163* expression indicates less atheroprotection. Both mechanisms may play a synergic effect on the onset of premature atherosclerosis in FH patients.

## Introduction

Familial hypercholesterolaemia (FH) is an inherited disorder characterized by high concentration of plasma low‐density lipoprotein (LDL) cholesterol levels, as result of mutations in the LDL receptor (LDLR) that impair liver LDL clearance 1, 2, and less frequently by genetic defects in the apolipoprotein B‐100 (Apo B) or the protease PCSK9 3. High LDL‐cholesterol (LDL‐c) levels lead to deposits of cholesterol in the arterial wall and promote atherosclerosis and the risk of premature coronary heart disease (CHD) 4. LDLR mutations include null or defective functions for ligand‐binding, transport, internalization and recycling 5, 6. Thus, LDLR mutations are a causal factor for atherosclerosis and the development of cardiovascular disease 7, 8, 9.

The atherosclerotic process, initiated by the accumulation of lipids particularly LDL in the subendothelial space, proceeds with activation of local inflammation and the innate immunity response 10. LDL retention by extracellular matrix proteoglycans favours its modification to form aggregates (agLDL) 11, 12. AgLDL is a potent inducer of massive intracellular cholesteryl ester (CE) accumulation in macrophages (foam cells) 13, 14, 15, 16. Indeed, macrophages are the main innate immunity cellular component of atherosclerotic plaques, leading to the proinflammatory response 17, 18. In atherosclerotic plaques, there are at least two distinct subtypes of macrophages (M1 and M2) 19; the M2‐macrophage phenotype is characterized by a high cell surface expression of CD163 20, a scavenger receptor associated to oxidative stress resistance 21.

Several receptors have been shown to participate in the uptake of modified LDL by human macrophages including the LDL‐receptor‐related proteins LRP5 22 and LRP1 16 and scavenger receptors such as CD36 23, 24 and MARCO 25.

Intracellular free cholesterol (FC) accumulation triggers a decrease in cholesterol synthesis by 3‐Hydroxy‐3‐methylglutaryl coenzyme A reductase (HMGCR) and in LDLR expression 26, 27 by inhibiting the sterol‐regulatory element binding pathway (SREBP) 28. FC undergoes re‐esterification to cholesteryl fatty acids esters (the ‘foam’ of foam cells) by acyl‐CoA:cholesterol ester transferase (ACAT) 29.

Our group has shown that LRP5 is involved in monocyte differentiation into macrophage 30 and is up‐regulated in macrophages exposed to agLDL 22. *In vivo* analyses in *Lrp5* ^−/−^mice fed a hypercholesterolaemic (HC) diet showed that LRP5 deficiency increases the development of aortic atherosclerotic lesions, suggesting an atheroprotective role for LRP5 31, 32. LRP5 belongs to the LDL receptor superfamily and shares common motifs including LDLR type A repeats, EGF‐like domains and transmembrane anchors. Interestingly, we have shown that LRP1, another member of this family, is involved in agLDL smooth muscle cell uptake leading to an increase intracellular CE accumulation 33, 34 and alteration in the phenotype and functional capacity 35.

Because LDLR is down‐regulated due to the excessive cholesterol, lipid‐loaded cells in the plaque need to uptake cholesterol through additional receptors in order to internalize cholesterol and contribute to plaque progression. FH patients, with mutations in the LDLR gene affecting its function and life‐long exposure to high LDL levels, are at high cardiovascular risk (HCVR) and develop premature coronary artery disease 36. We have previously reported that FH patients have a significant activation of the leucocyte‐cell lineage with increased levels of circulating cell‐derived microparticles of monocyte origin containing activation markers 37 which might directly contribute to exacerbate a proinflammatory condition both in blood and the vessel wall, facilitating the cellular crosstalk and delivery of paracrine molecular effectors between cells.

Here, we have investigated the expression profile of the LDL‐receptor‐related proteins (LRPs) in peripheral blood leucocytes and monocyte‐derived macrophages of FH patients and their relation with their phenotype and function (lipid intake).

## Materials and methods

### Population study and experimental design

In this study, we investigated 205 individuals of the Familial Hypercholesterolaemia SAFEHEART Cohort 38, distributed in three independent substudies consisting of 60, 63 and 82 individuals, respectively, as shown in Figure S1. SAFEHEART is an open, multicentre, long‐term prospective cohort study including patients with clinical and genetic diagnosis of heterozygous FH 39, 40 and relatives with negative genetic testing for FH, without or with secondary hypercholesterolaemia. Baseline characteristics of the cohort population have been previously described 38. Information on sociodemographic, clinical and lipid‐lowering treatment (LLT) data was obtained at the inclusion in the cohort registry. All FH patients in the study had LDL‐c levels within the pathological range (>150 mg/dl). Patients included in substudy 1 were under 40 years of age and with very high fasting LDL‐c plasma concentrations according to international guidelines 41. LDL‐c levels were 227 ± 4.6 mg/dl in substudy 1; 160 ± 10.7 mg/dl in substudy 2 and 176 ± 6.8 mg/dl in substudy 3. A similar pattern was observed for total cholesterol (TC) that was significantly higher in patients of substudy 1 compared to those in substudies 2 and 3. On the contrary, plasma HDL cholesterol and triglyceride were within the normal range in all the groups included in the study (Tables S1–S3), independently of the genetic diagnostic for FH and of treatment with lipid‐lowering therapies. As shown in Figure S1, two substudies were focused on peripheral blood leucocytes (PBL) and the third substudy aimed at analysing macrophages derived from isolated monocytes (MACs) obtained from blood of FH patients and non‐FH controls. The PBLs used in substudies 1 and 2 and macrophages in the substudy 3 were always obtained from single different patients.

Neither the FH nor control groups included patients with pregnancy, sepsis or infections and with history of cancer or suspected cardiovascular events. The results of the study are presented in accordance with STROBE guidelines, and the study was approved by the Local Ethics Committee of the ‘Investigación Clínica Fundación Jimenez Diaz (CEIC‐FJD)’ (protocol's number: 01/09) and was conducted according to the Declaration of Helsinki, and a written informed consent was obtained from all participants prior to the study.

#### Studies in PBL: substudies 1 and 2

Substudy 1: A young cohort (mean age: 35 years old) of 60 individuals was investigated. FH patients were distributed in two groups (*N* = 20 in each group). A healthy control group was included for comparative purposes (*N* = 20). Each one of the groups included 10 men and 10 women. Baseline characteristics (Table S1) show that the groups were matched by age, gender and other demography parameters. Briefly, FH groups consisted in patients with or without lipid‐lowering treatment (FH‐LLT^+^, *N* = 20; FH‐LLT^−^ group, *N* = 20), but with very high LDL‐c levels in plasma (>180 mg/dl). The FH‐LLT^+^ group included FH cases randomly chosen among those with a stable lipid‐lowering treatment (LLT^+^) of at least 1 year before inclusion, according to clinical guidelines 40, 42. FH‐LLT^−^ referred to FH patients who did not receive any lipid‐lowering treatment over the same time period, but matched for LDL‐c levels similar to those from the FH‐ LLT^+^ group. Individuals in the control LLT^−^ group (*N* = 20) did not have LDLR mutations and their LDL‐c level was in the normal range (below 115 mg/dl). Except for total cholesterol (TC) and LDL‐c, the FH and control groups did not differ in other lipid parameters, such as HDL cholesterol (HDL‐c) or triglycerides.

Substudy 2: A subgroup of FH patients (FH‐AT; *N* = 37) with subclinical carotid and aortic atherosclerotic lesions previously evidenced by magnetic resonance imaging (MRI) 36, 43 and a subgroup of control patients (non‐FH) with secondary hypercholesterolaemia (sc‐HC; *N* = 26) were investigated. Sociodemographic and clinical characteristics of the FH‐AT and sc‐HC groups are summarized in Table S2. All patients in the FH‐AT and sc‐HC groups received lipid‐lowering treatment (statins) according to guidelines. The groups did not differ in the levels of TC, LDL‐c and HDL‐c, but the ratio TC/HDL‐c was higher in the FH‐AT group than in patients with secondary hypercholesterolaemia. In contrast, triglycerides plasma levels and the percentage of individuals with obesity were significantly lower in the FH‐AT subgroup.

#### Monocyte‐derived macrophages (MACs): substudy 3

MACs were obtained from an independent subgroup of 62 FH patients (31 men and 31 women) and 20 control individuals (11 men and 9 women) from the SAFEHEART Cohort. Mean age of the control and FH groups at inclusion was of 46.5 years. FH patients were characterized by LDL‐c levels between 120 and 300 mg/dl and control individuals had LDL‐c levels ranging from 97 to 145 mg/dl (Table S3). Freshly isolated monocytes were differentiated into MACs as described below.

### Blood collection and sampling

Blood samples were withdrawn from the cubital vein without tourniquet using a 20‐gauge needle after 10–14 hrs of fasting. For the obtention of peripheral blood mononuclear cells (PBMN), blood samples were collected with a BD Vacutainer CPT System (Becton Dickinson) containing sodium heparin as anticoagulant and a ficoll‐hypaque solution for cell separation. Within 2 hrs of collection, blood samples were centrifuged at 1500–1800 rcf (relative centrifugal force) and PBMN were obtained by differential density gradient, as described by the providers. PBL‐derived RNA was directly obtained from blood samples collected in PAXgene tubes and processed according to manufacturer's instructions. For biochemical and DNA analysis, blood samples were collected without anticoagulant or in EDTA‐containing tubes 37. All serum and plasma samples were processed identically within 60 min. after extraction, aliquoted and frozen at −80°C, until required for analysis. DNA was obtained from blood cells according standard procedures, using a commercial kit (QiAmp Blood DNA Mini Kit, Qiagen, Germany) 44.

### Biochemical and genotyping analysis

Enzymatic methods were used to measure serum total cholesterol (TC), triglycerides (TG) and HDL‐c. LDL‐c was calculated by Friedewald formula (LDL = TC – HDL – TG/5); C‐reactive protein (CRP) and glucose were quantified by standard laboratory methods, as previously described 45. Molecular genetic diagnosis of FH was made using a DNA microarray (LIPOchip, Progenika Biopharma; Derio, Vizcaya, Spain) 46 and capillary sequencing by multiplex PCR conditions and sequence reactions 47, 48. Negative samples for the DNA array or sequencing were also analysed for large deletions or insertions using an adapted quantitative fluorescent multiplex PCR methodology.

### Primary cultures of human macrophages (MAC)

PBMN obtained by Vacutainer CPT System were suspended in RPMI‐1640‐glutamax culture media supplemented with 10% (vol:vol) human serum (group AB blood, Lonza; Basel, Switzerland), 100 U/ml penicillin/streptomycin, 2 mM L‐glutamine, 10 mM Hepes buffer (Gibco; Thermo Fisher Scientific corporation: Waltham, MA, USA) (HS‐media) and platted on 6‐well polystyrene plates (Becton Dickenson: Franklin Lakes, NJ, USA), to obtain a cellular density of 2 ×10^6^ monocyte/ml, as previously described 49. After incubation for 24 hrs, non‐adherent cells were removed by gently washing and adherent monocytes were allowed to differentiate into macrophages (MAC) for 7 days 50 changing the media every 48 hrs. Thereafter, cells were incubated for further 24 hrs in RPMI medium with 0.5% HS (minimal HS media). MACs were incubated in minimal‐HS medium with/without 100 μg/ml agLDL, for 24 hrs according to previous studies of our group 16, 22. Cells were then washed with phosphate buffer (PBS) and collected for RNA extraction. In specific subsets of experiments, LRP5 expression in human MAC was silenced with a commercial siRNA (Silencer^®^ siRNA ID No: s8293, Ambion; Invitrogen; Thermo Fisher Scientific corporation: Waltham, MA, USA). Briefly, human macrophages obtained from buffy coats were transfected with HiPerfect Transfection Reagent (Qiagen) according to the manufacturer's instructions. After 24 hrs, cells underwent the specified treatments and were collected. Silencer Selective negative control siRNA (Ambion) was used as a control and did not exert any effect on *LRP5* mRNA expression.

### LDL sample preparation and modification

LDL (density 1.019–1.063 g/ml) was prepared by ultracentrifugation from pooled plasma‐EDTA of normocholesterolemic volunteers 51. LDL‐protein concentration was determined using the bicinchoninic acid (BCA) method (Pierce Chemical Company; Thermo Fisher Scientific corporation: Waltham, MA, USA) and LDL purity assessed by agarose‐gel electrophoresis (SAS‐MX Lipo‐kit, Helena Biosciences:, Gateshead, UK). LDL preparations were tested to exclude the presence of endotoxin (Limulus amebocyte lysate test, BioWhittaker: Walkersville, MD, USA) and proved to be negative in all cases. LDL used in the experiments was less than 48 hrs old. AgLDL were generated by vortexing LDL (1 mg/ml) according to the method previously described by Guyton *et al*. 52 and as previously performed by our group 33. This method has shown to produce similar LDL aggregation as LDL versican incubation 53. In all experiments, LDL oxidation (before and after aggregation) was excluded by assessing thiobarbituric‐acid‐reactive substances (TBARS) formation, according Ohkawa *et al*. 54 with slight modifications 49.

### Macrophage lipid staining

Human macrophages, treated with/without 100 μg/ml agLDL in culture, were washed with PBS and fixed with 4% paraformaldehyde. Thereafter, cells were progressively dehydrated with alcohol and stained with Herxheimer's solution and Mayer's haematoxylin. Images were obtained with an inverted microscopy ECLIPSE TS100 (Nikon; Tokyo, Japan) with ×300 magnification.

For quantification of lipid internalization, human macrophages incubated with/without agLDL were fixed with 4% paraformaldehyde, permeabilized with 0.5% Tween and incubated in blocking buffer (3% bovine serum albumin in PBS) before adding 5 μl/ml medium of DiI (30 mg/ml in DMSO, Sigma‐Aldrich) for 1 hr, washed and covered with Prolong Gold antifade reagent. Images of labelled cells were recorded on a Leica inverted confocal microscope (Leica TCS SP2‐AOBS; Leica: Wetzlar, Germany) using the HCX PL APO 63x/1.2W Corr/0.17 CS objective. Images were acquired in a scan format of 1024 × 1024 pixels in a spatial data set (xyz) and processed with the Leica Standard Software TCS‐AOBS.

### Murine model

Homozygous wild‐type C57BL/6 mice and *Lrp5* ^*−/−*^ C57BL/6 mice were used in the study. *Lrp5* ^−/−^ mice, a kind gift from Dr. Bart Williams 55, were maintained in a C57BL/6 background. The animals housed in cages under controlled temperature (21 ± 2°C) on a 12 hrs light/dark cycle with food and water *ad libitum*. At 10 weeks of age, animals were divided into two groups to be fed with normal chow diet (NC) or with high‐cholesterol diet (HC, TD.88137, Harland Labs, Indianapolis, IN, USA) for further 8 weeks (7 mice/group). Composition of lipid profile was analysed as previously described 32. At the end of the treatment period, blood was obtained by cardiac puncture under terminal anaesthesia (1 mg/kg Medetomidine and 75 mg/kg Ketamine, ip), before surgical excision. Blood samples (400 μl) were collected in PAXgene tubes to extract blood‐derived mRNA. Thereafter, aortas were dissected and carefully cleaned of adventitial tissue under a stereoscopy microscopy. All procedures fulfilled the criteria established by the ‘Guide for the Care and Use of Laboratory Animals’, and the study protocol was approved by the Hospital de la Santa Creu i Sant Pau Animal Research Committee (ICCC051/5422).

### Real‐time PCR analysis

Total RNA from human macrophages was extracted using the mirVana miRNA Isolation Kit (Ambion). RNA extraction from PBL of human and mice samples was performed with the PAXgene blood RNA kit (PreAnalytiX, Qiagen/Becton Dickenson), according to manufacturer's instructions. RNA concentration was determined with a NanoDrop ND‐1000 spectrophotometer (NanoDrop Technologies; Thermo Fisher Scientific corporation; Waltham, MA, USA), and purity was checked by the A260/A280 ratio. Reverse transcription of genes was performed with the High Capacity cDNA Reverse Transcription Kit followed by Taqman real‐time PCR amplification, according to the manufacturer's instruction (Applied Biosystems). Genes expression were analysed by specific primers (Applied Biosystems: Foster City, CA, USA) for LRP5 (Hs00182031_m1; Mm01227476_m1), LRP1 (Hs00233856_m1; Mm00464608_m1), LDLR (Hs01092524_m1; Mm01177349_m1), HMGCR (Hs00168352_m1; Mm01282499_m1), LRP6 (Hs00233945_m1; Mm00999795_m1), CD163 (Hs00174705_m1; Mm00474091_m1), CD36 (Hs01567185_m1) and MARCO (Hs00198935_m1). Samples were analysed in duplicates, and only mRNAs with expression levels below 32 cycles were accepted. PIK3C2A (Hs00153223_m1) and 18S‐RNA (4333760F) were used as endogenous internal controls to normalize the mRNA expression levels for human 49 and mouse samples, respectively. Data were analysed by SDS 2.4, RQ Manager 1.2.1 and DataAssits v3.0.1 software. (Applied Biosystems: Foster City, CA, USA).

### Immunohistochemistry

Serial sections (5 μm thick) of paraffin‐embedded mouse aortas were placed on poly‐L‐lysine‐coated slides, deparaffinized and treated with H_2_O_2_ for suppression of endogenous peroxidase activity. Immunohistochemical analysis was performed with the avidin‐biotin immunoperoxidase technique, as previously described 56. Samples were analysed for Apo B (rabbit‐polyclonal anti‐apolipoprotein B; dilution 1:100; Abcam, ab20737, Abcam: Cambridge, UK), LRP5 (rabbit‐polyclonal anti‐LRP5; dilution 1:50; Abcam, ab38311) and MAC387 that is a marker for tissue infiltrating monocytes 57, 58 [anti‐Macrophage antibody (MAC387); dilution 1:100; Abcam, ab22506]. Images were captured and digitized using a Nicon Eclipse 80i microscope and a Retiga 1330i Fast camera. Controls without primary antibodies were run with each set of specimens and showed no labelling background.

### Determination of free and esterified cholesterol content

siRNA‐LRP5 and siRNA‐random treated human MACs were incubated for 8 hrs with 100 or 400 μg/ml agLDL. Cells were serially washed with PBS, PBS/1% BSA and PBS/1%BSA/heparin 100 U/ml and harvested into NaOH 0.1N. Lipid extraction and thin‐layer chromatography were performed as previously described 59, 60. The spots corresponding to free cholesterol (FC) and cholesteryl esters (CE) were quantified by densitometry against a standard curve of cholesterol and cholesterol palmitate, respectively, with the use of a computing densitometer (Molecular Dynamics: Sunnyvale, CA, USA).

### Statistical analysis

Data are expressed as mean ± SEM (standard error of the mean), except when indicated. Minimal required sample size was calculated according Noordzij *et al*. 61. A test for normality was performed using Shapiro–Wilk test. Outlier values were excluded by Chauvenet's criterion. The statistical significances between two groups were determined with Mann‐Whitney U‐test and multiple comparisons by Kruskal–Wallis test, and when significant, Bonferroni post hoc analysis was used to assess intergroup differences. Categorical variables (gender, risk factors and medication) were compared using a Chi‐square analysis of frequencies. Strength of the association between continuous variables was calculated by Spearman's correlation coefficients. Statistical analysis was performed with Stat View 5.0.1 (Abacus concepts; Piscataway, NJ, USA) and SPSS Statistics Version 21.0.0 (SPSS, Chicago, IL, USA). A *P* ≤ 0.05 was considered statistically significant.

## Results

### LRP expression levels in PBL from FH patients

To explore whether transcriptional levels of cell‐membrane receptors involved in LDL uptake are affected in the PBL fraction of FH patients, we analysed mRNA ‐expression of *LRP1*,* LRP5*,* LDLR* and *HMGCR* by real‐time PCR in the three groups of young individuals (control non‐FH‐LLT^−^, FH‐LLT^−^ and FH‐LLT^+^), with an average age of 35 years. As shown in Table 1, *LRP5* gene expression level was higher in FH‐LLT^−^ patients compared with controls (control LLT^−^, *P* = 0.040). In contrast, controls and FH‐LLT^+^ patients showed similar results. PBL from FH‐LLT^−^ patients showed higher *LRP5* gene expression than those from the FH‐LLT^+^ group (*P* = 0.016), although LDL‐c levels did not differ significantly between FH‐LLT^+^ and FH‐LLT^−^ groups (groups matched for this variable). Similarly, PBL of FH‐LLT^−^ patients showed an increased expression for *LRP1* (*P* = 0.044) when compared with FH‐LLT^+^. Differences in LRP1 levels between FH‐LLT^−^ and healthy controls did not achieve statistical significance. *LDLR* and *HMGCR* gene expression levels were significantly decreased in PBL from FH patients with respect to the healthy controls, independently of the lipid‐lowering therapy (FH‐LLT^−^: *LDLR*,* P* = 0.043 and *HMGCR*,* P* = 0.034; FH‐LLT^+^: *LDLR*,* P* = 0.020 and *HMGCR*,* P* = 0.012).

**Table 1:** Expression level of LDL receptors and HMGCR in peripheral blood leucocyte (PBL) of young FH patients and healthy matched controls

Genes	Control LLT^−^	FH‐LLT^−^	FH‐LLT^+^
*LRP1*	78.7 ± 3.0	90.3 ± 5.8^b^	77.5 ± 3.5
*LRP5*	74.2 ± 2.3	82.4 ± 2.9^a^^,^ b	73.6 ± 2.9
*LDLR*	90 ± 4.1	77.1 ± 4.2^a^	75.3 ± 4.6^a^
*HMGCR*	11.7 ± 0.6	9.8 ± 0.5^a^	9.7 ± 0.6^a^

Gene expression levels in PBL. Control LLT^−^ *versus* FH‐LLT^+^ and FH‐LLT^−^ group. *N* = 20/group. Real‐time PCR analysis normalized to *PIK3C2A* by calibration curve. Results are shown as mean±SEM in relative units ×100.

a *P* < 0.05 *versus* Control.

b *P* < 0.05 *versus* FH‐LLT^+^.

In addition, levels of LRP5, but not *LRP1*, were significantly higher in FH patients (FH‐AT group) than in non‐FH patients (sc‐HC) (Table 2). In addition, the FH‐AT group had significantly higher *LRP6* levels compared with their hypercholesterolaemic non‐FH controls. LRP6 is a protein with high homology to LRP5 and both receptors carry out redundant functions 62. Overall, *LRP5* expression levels in PBL were significantly higher in FH patients (*N* = 77) than in the non‐FH (*N* = 46) (86.8 ± 2.3 *versus* 78.8 ± 2.8, *P* = 0.019). Therefore, LRP5‐dependent signalling pathways are increased in the leucocytes of patients with FH.

**Table 2:** Expression level of low‐density lipoprotein receptor‐related protein in (PBL) in MRI characterized FH patients and hypercholesterolaemic non‐FH matched controls

Genes	sc‐HC	FH‐AT
*LRP1*	72.7 ± 3.9	78.8 ± 3.2
*LRP5*	81.9 ± 4.4	95.1 ± 3.5^a^
*LRP6*	85.2 ± 5.0	109.5 ± 5.6^a^

Gene expression levels in PBL. sc‐HC (*N* = 26) *versus* FH‐AT (*N* = 37). Real‐time PCR analysis normalized to *PIK3C2A* by calibration curve. Results are shown as mean±SEM in relative units ×100.

a *P* < 0.05 *versus* sc‐HC.

### *LRP5* expression levels and atherosclerotic burden in FH patients

Overexpression of *LRP5* is found in infiltrated macrophages of human advanced coronary atherosclerotic plaques 22, and *LRP1*‐overexpression has been reported in atherosclerotic lesions both in animal models and human lesions 63, 64, 65, 66. Interestingly, in PBL of FH patients with atherosclerotic lesions (FH‐AT), the expression level of transcripts for *LRP1*,* LRP5* and *LRP6* was similar in patients with atherosclerotic burden in one or more arterial beds (Fig. S2).

### *LRP5* is up‐regulated in monocyte‐derived macrophages from FH patients after agLDL exposure

**Figure 1:**
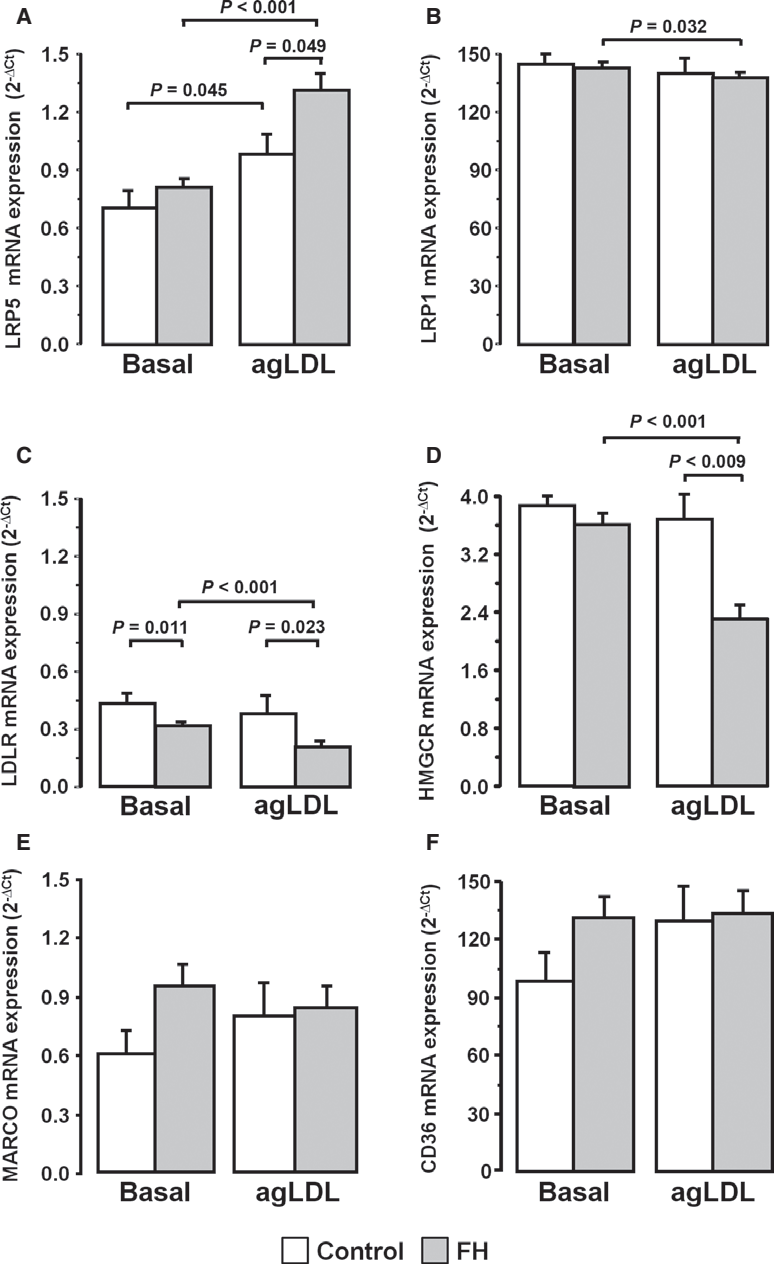
Increased ***LRP5*** expression level in macrophages from control and FH patients. Macrophages were incubated with 100 μg/ml agLDL for 24 hrs. The gene expression of *LRP1* **(A)**,* LRP5* **(B)**,* LDLR* **(C)**,* HMGCR* **(D)**,* MARCO* **(E)** and *CD36* **(F)** in agLDL unloaded (Basal) and loaded macrophages (agLDL). Control group (white bars, *N* = 20) and FH patients (grey bars, *N* = 62). Bars represent mean±SEM of expression level for both groups in duplicated. Only *P* values < 0.05 are shown.

Next, we investigated if cell‐membrane receptors participating in the uptake of LDL are differently expressed in macrophages from FH patients. To this aim, *LRP1*,* LRP5*,* LDLR* and *HMGCR* were analysed by real‐time PCR in agLDL‐loaded macrophages of control (healthy individuals, *N* = 20) and FH patient (*N* = 62). At baseline, expression levels of *LRP5* did not differ significantly between macrophages obtained from controls and FH patients (Fig. 1A); however, FH‐MACs showed a significant increase in *LRP5* gene transcription when exposed to agLDL (1.63‐fold increase; *P* < 0.001), an effect that was also found, although with lower intensity level, in control MACs (1.40‐fold increase; *P* = 0.045). The induction of *LRP5* by agLDL was significantly higher in FH‐MACs than in control MACs (1.35‐fold, *P* = 0.049).

*LRP1* transcription levels in human macrophages were much higher than those of *LRP5*, and they were minimally affected by exposure to 100 ug/ml agLDL. In FH‐MACs, *LRP1* expression levels were slightly, but significantly, reduced from baseline value (7%, *P* = 0.032) in LDL‐exposed cells (Fig. 1B). *LDLR* mRNA expression level in FH‐MACs were 26% lower than in control MACs (*P* = 0.011). *LDLR* transcription level was further reduced in FH‐MACs after exposure to agLDL (1.5‐fold, *P* < 0.001) (Fig. 1C), effect that was not observed in macrophages from healthy individuals. Similarly, *HMGCR* mRNA expression level was significantly decreased in FH‐MACs (1.56‐fold, *P* < 0.001) after exposure to agLDL (Fig. 1D). In the presence of agLDL, transcription levels of both *LDLR* and *HMGCR* were significantly reduced in FH‐MACs when compared with non‐FH‐MACs (45%, *P* = 0.023 and 40%, *P* = 0.009, respectively). To examine whether the FH background affected the response to agLDL of scavenger receptors, *MARCO* and *CD36,* as representative class A and class B scavengers associated with development of atherosclerotic lesions, were analysed. As shown in Figure 1E and F, neither control MACs nor FH‐MACs showed statistically significant changes in *MARCO* and *CD36* mRNA‐expression levels after 24‐hr exposure to agLDL.

### Lipid staining in macrophages of patients with FH

**Figure 2:**
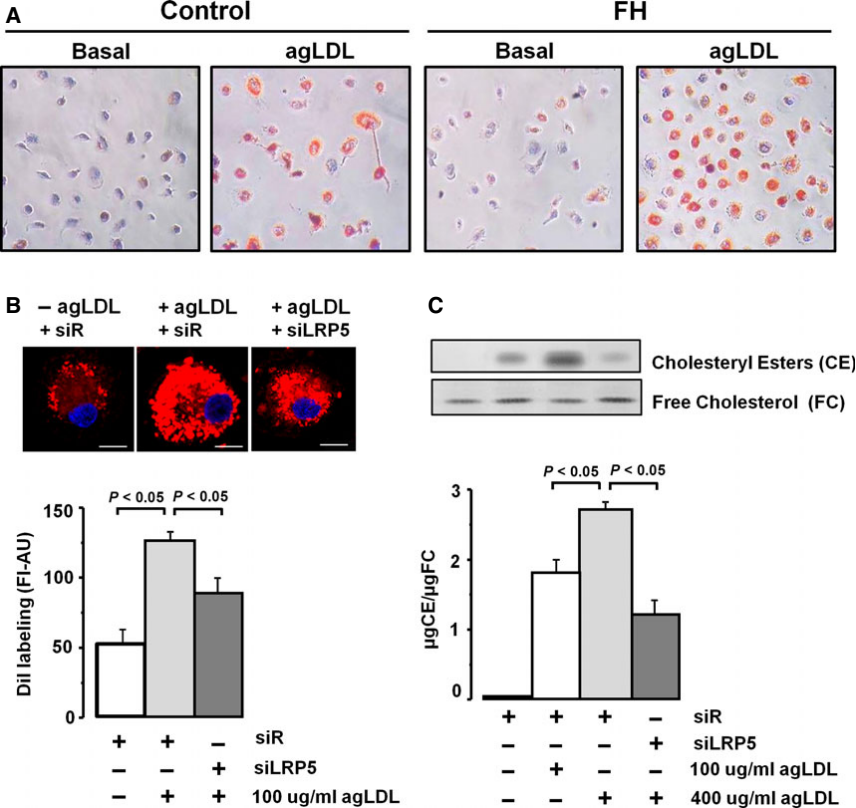
Internalization of agLDL in macrophages. **(A)** Lipid inclusion showed by red colour into macrophages from controls and FH patients, by Herxheimer's stain. Images are representative. **(B)** Confocal microscopy of LRP5‐silenced human macrophages and treated with 100 μg/ml agLDL for 8 hrs, washed, fixed and stained with DiI. Fluorescence intensity was measured with the Leica Standard Software TCS‐AOBS (*N* = 28–32 cells/condition). Bar: 10 μm. *N* = 3. **(C)** Human macrophages transfected to siRNA‐Random (siR) or siRNA‐LRP5 (siLRP5) were exposed to agLDL during 8 hrs. Macrophages were then exhaustively washed and harvested to measure intracellular cholesteryl esters (CE) and free cholesterol (FC) by thin‐layer chromatography. Bar graph show quantitative values of CE relative to FC. *N* = 3.

Internalization of 100 μg/ml agLDL was analysed in control MACs and FH‐MACs by staining for total lipids. In control conditions, without agLDL loading, the lipid content was negligible in control MACs and had a low intensity stain in FH‐MACs. In contrast, when agLDL was added to the medium, there was a strong lipid staining in both control and FH‐MACs (Fig. 2A). These results show that FH**‐**MACs retain the capability to internalize LDL, a process that in these cells that have down‐regulated expression of LDLR may be produced by overexpression of *LRP5*.

### LRP5 silencing decreases CE accumulation after agLDL treatment in human macrophages

To proof that LRP5 is involved in intracellular cholesterol accumulation, siRNA‐LRP5 and random siRNA‐treated control MACs were incubated with agLDL and labelled with Dil. As shown in Figure 2B, cells exposed to agLDL showed a strong vesicular DiI labelling (fluorescence signal >2‐fold higher than without LDLs); however, the intensity of DiI labelling was significantly reduced in MACs with silenced *LRP5* expression indicating a reduced uptake of LDL in LRP5‐silenced MACs (*P* < 0.05 *versus* controls). Analysis of intracellular esterified (CE) and free (FC) cholesterol by thin‐layer chromatography (Fig. 2C) further demonstrated that silencing of *LRP5* interferes with lipid uptake in MACs. Thus, exposure of macrophages to agLDL induced a dose‐dependent intracellular CE accumulation (100–400 μg/ml agLDL), whereas silencing *LRP5* expression significantly reduced CE internalization (*P* < 0.05 *versus* non‐LRP5 silenced cells) in MACs exposed to 400 μg/ml agLDL. In summary, LRP5 internalizes cholesterol in human MACs.

### AgLDL affects *LRP5* expression in FH macrophages independently of LDLR expression

The heterogeneity of *LDLR* mutations among FH patients in the SAFEHEART cohort and the predicted effects of the different mutations on *LDLR* functionality are listed in Table S4. Patients with mutations entailing a shift reading frame (*N* = 10) or mutations entailing an early stop codon leading to lack of protein (*N* = 12) were included in the FH‐LDLR‐null group (FH null; *N* = 22), whereas the FH‐LDLR‐non‐null group consisted of patients (FH non‐null; *N* = 40) with mutations on the promoter (*N* = 4), mutations entailing an amino acid change (*N* = 15), mutations with a splicing change (*N* = 9) and other mutations inducing the production of a mutated protein (*N* = 12). At baseline, FH non‐null and FH null groups did not differ in the expression levels of the LRPs (*LRP5, LRP1*) neither in the expression of scavenger receptors (*MARCO*,* CD36*) (Fig. S3). *LRP5* expression levels were increased 1.78‐fold (*P* < 0.001) and 1.63‐fold (*P* = 0.023) in MACs from non‐null and null patients, respectively, in the presence of agLDL (Fig. S3A). The degree of the increase did not relate to the severity of the gene mutation (null *versus* non‐null, *P* = 0.446). Interestingly, *LRP1* expression level was significantly reduced in lipid‐loaded macrophages of FH non‐null patients (91% of baseline value: *P* = 0.012), whereas this effect was not found in the FH null group (Fig. S3B). On the contrary, the transcriptional pattern of *MARCO* and *CD36* in response to agLDL did not differ significantly between the FH non‐null and the FH null groups (Fig. S3C and D).

### *LRP5* is highly expressed in macrophages of FH patients with low *LDLR* levels

**Figure 3:**
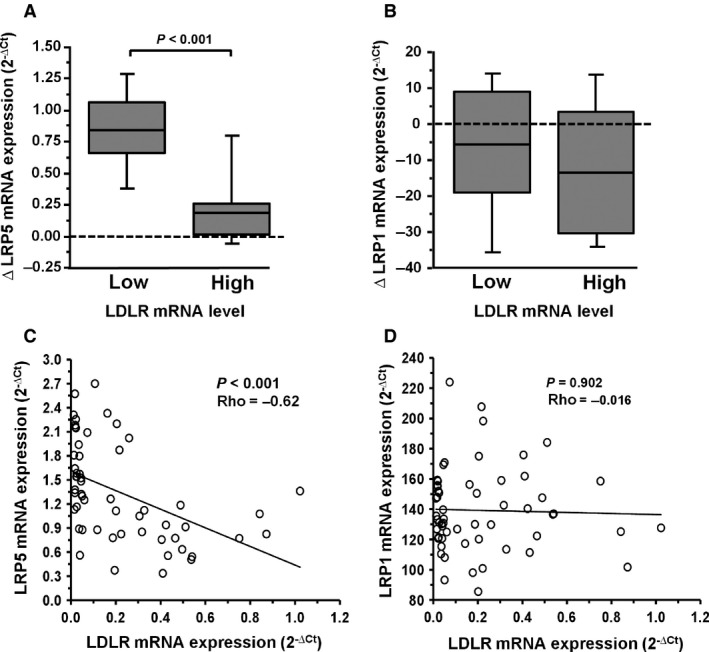
Relationship between ***LRP**5* and ***LDLR*** in FH macrophage. Expression levels analysed by real‐time PCR quantification for *LRP5*,* LRP1* and *LDLR* from Macrophages (*N* = 62) incubated with or without 100 μg/ml agLDL for 24 hrs. The box plot was designed with increasing expression (∆, agLDL – Basal) for *LRP5* **(A)** and *LRP1* **(B)**. *LRP5* and *LRP1* expression levels associated with high or low values respect to the median for *LDLR* (0.112), as cut‐off. ∆ *LRP5 versus* low *LDLR* values (median and IQR: 0.85 and 0.39; mean±SEM: 0.83 ± 0.08) and ∆ *LRP5* expression level *versus* high *LDLR* values (median and IQR: 0.18 and 0.24; mean±SEM: 0.22 ± 0.08). ∆ *LRP1 versus* low *LDLR* levels (median and IQR: −5.77 and 28.08; mean±SEM: −6.89 ± 3.84) and ∆ *LRP1 versus* high *LDLR* values (median and IQR: −13.35 and 33.73; mean±SEM: −11.83 ± 3.79). IQR: Interquartile range. Correlation between *LRP5 versus LDLR* **(C)** and *LRP1 versus LDLR* **(D)**. The *P*‐values were obtained by Spearman's correlation (ρ). Gene expression was performed in duplicated. Only *P* values < 0.05 are shown.

FH‐MACs with *LDLR* gene expression below and above the median value as cut‐off level (mRNA level [2^−∆Ct^]: 0.112) showed different *LRP5* expression pattern in response to agLDL. As shown in Figure 3A, the increase in *LRP5* mRNA expression due to agLDL internalization was significantly more evident in those cells obtained from patients with lower *LDLR* levels. Contrarily, changes to baseline in *LRP1* gene expression after exposure of macrophage to agLDL did not relate to the LDLR levels (Fig. 3B). In addition, there was a negative correlation between *LRP5* and *LDLR* gene expression levels in lipid‐loaded macrophages from FH patients (Fig. 3C), whereas no significant correlation was found between *LRP1* and *LDLR* mRNA expression levels (Fig. 3D). In summary, LRP5 seems to substitute LDLR in uptaking lipids in macrophages of FH patients.

### *CD163* expression is down‐regulated in macrophages of FH patients

**Figure 4:**
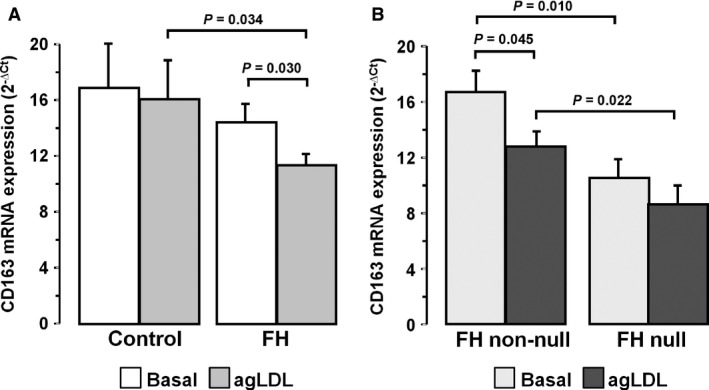
Decreased ***CD16**3* expression level in macrophages of FH patients. Differential expression level in lipid‐loaded macrophages from controls (*N* = 20) and FH patients (*N* = 62) **(A)** and FH genotype: FH non‐null (*N* = 40) and FH null (*N* = 22) **(B)**. Bars represent mean±SEM of expression level in duplicated. Only *P* < 0.05 are shown.

We have recently shown that *LRP5* expression in human macrophages associates with the patrolling/non‐inflammatory CD16^+^ phenotype (M2‐macrophages) 67. Here, we investigated whether FH affects the expression of the scavenger receptor CD163, also associated to the M2‐macrophage phenotype. *CD163* expression level did not show significant changes in control MACs exposed to agLDL (*P* = NS), but it was significantly decreased in FH‐MACs (11.29 ± 0.89 *versus* 14.51 ± 1.16, *P* = 0.030). Furthermore, *CD163* showed a significantly lower expression in FH‐MACs than in control MACs in the presence of agLDL (1.42‐fold decrease, *P* = 0.034) (Fig. 4A). In FH patients, *CD163* transcription decreased in the most severe forms of *LDLR* mutation (% decrease FH null *versus* FH non‐null: Basal, 38%, *P* = 0.010 and agLDL, 32%, *P* = 0.022). FH non‐null patients showed a statistically significant decrease in *CD163* expression by exposing macrophages to agLDL (25% decrease, *P* = 0.045) (Fig. 4B). In summary, these results indicate that macrophages of FH patients have less capacity to elicit CD163‐mediated anti‐inflammatory responses in the presence of high LDL levels.

### *LRP5* expression is up‐regulated in PBL of hypercholesterolaemic mice

**Figure 5:**
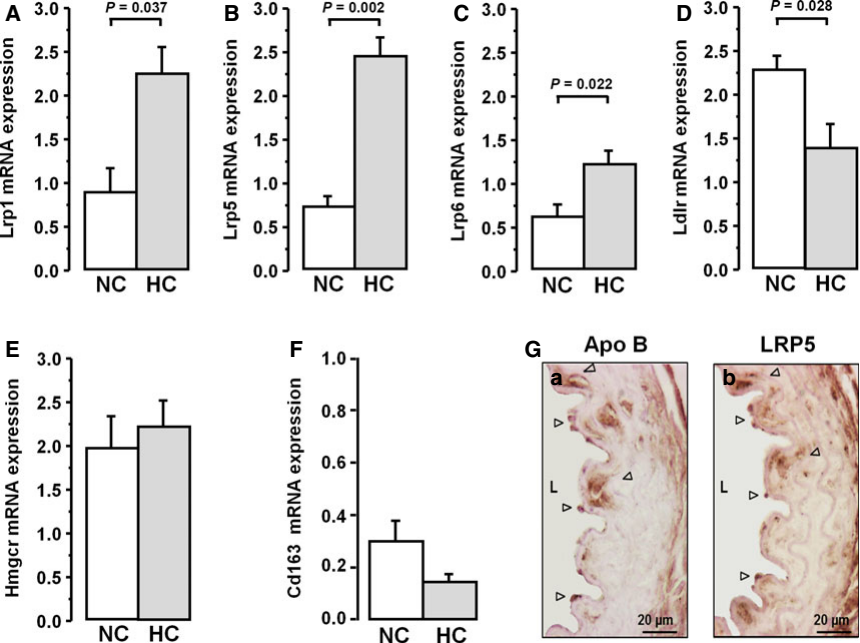
Hypercholesterolaemic effect on LRP5 in the murine model. Gene expression levels of *Lrp1* **(A)**,* Lrp5* **(B)**,* Lrp6* **(C)** *, Ldlr* **(D),** *Hmgcr* **(E)** and ***Cd163*** **(F)** in PBL from mice fed a normocholesterolemic (NC) and a hypercholesterolaemic (HC) diet analysed by real‐time PCR and normalized to 18S. Bars represent mean±SEM of expression level in duplicated. Only *P* values < 0.05 are shown. **(G)** Representative images of mice aortas (HC) labelled for Apolipoprotein B (Apo B) and LRP5. *N* = 7 mice/group. Arrow point shows the positive labelling. L: lumen.

We have shown the increased expression of *LRP5* in PBLs of FH patients that have been exposed to a life‐long exposure to high LDL levels due to mutations in the LDLR gene. Here, we induced hypercholesterolaemia in homozygous wild‐type C57BL/6 mice (Wt) and in *Lrp5* ^*−/−*^ C57BL/6 mice by feeding a cholesterol‐rich diet. We analysed PBL‐gene expression of *Lrp1*,* Lrp5*,* Lrp6*,* Ldlr, Hmgcr* and *Cd163*. Wt‐mice fed a high‐cholesterol diet (HC) for 8 weeks showed a significant but moderated increase in plasma TC respect to normocholesterolemic (NC) animals (∆TC = 62.5 mg/dl, *P* < 0.005) 32. Gene expression levels of LRPs were increased in PBL of HC mice (Fold change *Lrp1*: 2.0‐fold, *P* = 0.037; *Lrp5*: 3.30‐fold, *P* = 0.002, *Lrp6*: 2.0‐fold; *P* = 0.022). High cholesterol levels induced down‐regulation of *Ldlr* (*P* = 0.028), as expected, and *Hmgcr* gene expression levels were not significantly changed (Fig. 5A–E). In addition, PBLs in the HC group showed 2‐fold decrease in the mean *Cd163*‐expression level, although the difference with the NC‐group did not achieve significance (Fig. 5F).

In contrast to Wt‐mice, *Lrp5* ^*−/−*^ animals showed a high increase in plasma TC after 8 weeks under hypercholesterolaemic diet when compared with NC‐ *Lrp5* ^*−/−*^ mice (∆TC = 140.0 mg/dl, *P* < 0.005) 32. Besides, *Cd163* was significantly increased in LRP5‐deficient (*Lrp5* ^*−/−*^) mice compared with the Wt in both normocholesterolemic and hypercholesterolaemic animals (>2‐fold increase; *P* < 0.049; Fig. S4).

To determine if the increased *Lrp5* gene expression levels observed in cultured MACs were also observed *in vivo*, we performed immunohistochemistry analysis of mice aortas at the proximal side of the aortic arch, the area with the strongest staining for lipids using Oil‐red‐O (ORO) in the hypercholesterolaemic mice (data not shown). As shown in Figure 5G, Apo B infiltration was evident in the aorta of mice fed a hypercholesterolaemic diet (brown signals in Fig. 5G‐a). Immunostaining for LRP5 in adjacent sections of the aorta depicted a similar positive distribution pattern, especially at the lumen side in areas underlying the endothelium with adhered monocytes (MAC387 positive cells), that were strongly positive for Apo B and LRP5 (compare Fig. 5G‐a and b).

## Discussion

Severe hypercholesterolaemia is a known causal factor for atherosclerosis and cardiovascular disease 68, caused by genetic defects in the catabolism of LDLs, leads patients to long‐term exposure of high plasma cholesterol levels and consequently to high risk of presenting coronary heart disease and its clinical manifestation of myocardial infarction at young age.

Atherosclerosis is a chronic inflammatory process that involves LDL infiltration and activation of the innate immunity. It is characterized by the development of lipid‐rich lesions in the arterial wall, in which monocyte‐derived lipid‐laden macrophages are frequently found. LRPs are present in human atherosclerotic lesions in association with VSMC and macrophages 16, 22, 69. Here, we provide evidence that bloodstream PBL in young heterozygous FH patients with genetically characterized LDLR mutations have higher expression of *LRP5* than age‐matched healthy controls or patients with secondary hypercholesterolaemia. This increase went along with an increase in its highly homologous receptor *LRP6*. Experimental studies in mice models selectively lacking either LRP5 or LRP6 suggest an overlapping of their biological roles 62. Differences in the sensitivity to plasma LDL levels might account for the lower response of *LRP1* mRNA expression to the hypercholesterolaemic background in our heterozygous FH patients. This might explain the apparent discrepancy between our findings and those reported by Mosig *et al*. 70, 71 about the increasing expression of LRP1 in blood isolated monocytes of FH patients with high degree of disease severity (50% patients with a homozygous pattern). In this respect, gene transcription changes in PBL were more evident for *Lrp5* (>3‐fold) than for *Lrp1* (2‐fold) in our diet‐induced hypercholesterolaemic mice model.

A key pathogenic event in the development of atherosclerosis is the retention of lipoprotein particles in the vessel wall where they are aggregated and anchored to proteoglycans of the subendothelial matrix 35, 72. Macrophages attempt to clear the particles and become filled with esterified cholesterol droplets. We and others have demonstrated that LRPs play a crucial role in the uptake of agLDL either in vascular smooth muscle cells or in macrophages 33, 73. Moreover, our group has reported a higher efficacy of LRP5 than LRP1 for promoting CE accumulation in human macrophages 22 which was related to an early saturation of LRP1 lipid uptake activity. Here, expanding these previous findings, we provide evidence that the exposure of macrophages to agLDL for prolonged periods of time results in a transcriptional up‐regulation of *LRP5* but not of *LRP1*, effect that was more evident in FH patients than in healthy individuals. It is interesting to note that the agLDL‐induced up‐regulation of *LRP5* was significantly larger in FH patients with lower *LDLR* expression levels, an effect that was not observed for *LRP1*.

The relevance of other LDL‐binding receptors for the uptake of agLDL in macrophages has not yet been well defined. In this study, macrophage scavenger receptors as *MARCO* or *CD36* did not show any consistent changes at the transcriptional level in response to agLDL and to the level of intracellular CE, either in FH or in healthy volunteers. These findings strongly reinforce the relevance of LRP5 in maintaining the capacity of monocytes and macrophages to internalize LDL in FH patients and therefore promote lipid clearance from the arterial intima and eventually contributing to plaque formation when the pathways gets saturated. In line with these results, diet‐induced hypercholesterolaemic mice showed a strong colocalization of Apo B and LRP5 positive signals within the arterial intima as well as in monocytes adhered to the vascular endothelium. The importance of LRP5 in agLDL internalization in human macrophages is supported by the finding that LRP5 silencing results in low intracellular lipid accumulation and a reduced CE content. However, the contribution of LRP5 to atherosclerosis is still not well defined and its function may depend on cell lineage. The contribution of LRP5 to cholesterol metabolism was previously suggested by the observation that *ApoE* ^*−/−*^ *LRP5* ^*−/−*^ mice have larger atherosclerotic lesions than their *ApoE* ^*−/−*^ littermates 74. Beyond its role in promoting lipid internalization, increased expression of LRP5 also induces the activation of canonical Wnt signalling 22, 31. In macrophages, canonical Wnt signalling is thought to promote cell motility through a β‐catenin‐dependent mechanism 75. Using a systems biology approach, Ramsey *et al*. reported the up‐regulation of the canonical Wnt signalling downstream effector β‐catenin within macrophage‐rich regressing plaques in two mechanistically distinct lipid‐lowering mouse models of plaque regression 76. Supporting these finding in animal models, human *LRP6* genetic variants that impair Wnt/β‐catenin signalling have been associated with increased risk of carotid atherosclerosis 77 and early coronary artery disease 78 in humans, suggesting protective and anti‐atherogenic effects associated to canonical Wnt/β‐catenin signalling.

Blood monocytes recruited and infiltrated in the arterial intima differentiate into various subtypes of macrophages that are characterized by their surface epitopes and cell‐membrane receptors. In a recent study, we have reported a significantly higher LRP5 expression in macrophages derived from CD14^+^CD16^+^ patrolling monocytes than in those derived from pro‐inflammatory CD14^+^CD16^−^ monocytes 67. We have also shown that a higher number of monocytes differentiate towards a CD16^+^ phenotype in the presence of native LDL 49. Macrophages CD16^+^ are usually related to a mature M2‐phenotype, also characterized for being positive for CD163 20 a scavenger receptor of the haemoglobin–haptoglobin complex involved in haeme catabolism through a process mediated by regulation of the haeme oxygenase‐1 (HMOX1) expression 79. The presence of CD163 macrophages in human atherosclerotic lesions has been also associated with anti‐inflammatory properties mediated through IL10 21, and studies in mice *ApoE* ^*−/−*^ models have associated plaque regression with the presence of M2 macrophage expressing CD163 80, 81, 82. Although differentiation of monocytes into macrophages is likely to be terminal, macrophages have the ability to switch phenotype and functional characteristics in response to external signals. It is interesting to note that macrophages derived from monocytes of FH patients have decreased expression of *CD163* when exposed to agLDL, an effect that was not evident in macrophages from healthy individuals, suggesting a lower HMOX1 mediated atheroprotective potential in FH. Indeed, FH patients carrying a null‐mutation and therefore with a long‐life exposure to high LDL levels and a severe atherogenic background were those with lower *CD163* levels, both in the absence and in the presence of agLDL. In accordance, monocytes exposed to native LDL in atherogenic concentration during differentiation into macrophages also lose surface expression of CD163 49. Moreover, inverse translation studies in mice also showed a trend to lower levels *Cd163*‐PBL in diet induced mildly hypercholesterolaemic animals, and *Cd163*‐expression was highly up‐regulated in mice lacking *Lrp5*, suggesting a direct relationship between high *LRP5* and low *CD163* levels in macrophages of FH patients.

Taken in account the above results, we could hypothesize that higher levels LRP5 and lower levels of CD163 in arterial macrophages might account for the higher atherosclerotic burden in FH patients when compared with individuals within the same age interval and similar risk factors for atherosclerosis beyond the genetic diagnostic of FH 36.

In conclusion, our results show for first time that other members of the LDL receptor family such as LRP5 and LRP6 are highly active lipid internalization receptors in cells of the innate immunity system in FH patients. Macrophages derived from mononuclear cells that were formed and circulated in a niche of high LDL concentrations have down‐regulated LDLR but retain the LDL internalization function. LRP5 and LRP6 are the receptors responsible for lipid internalization in FH macrophages. FH macrophages seem to present lower anti‐inflammatory activity and less capacity of protection against LDL‐mediated oxidative stress due to their lower content in the scavenger receptor CD163, contributing therefore to the premature development of atherosclerosis in these patients. Further studies are warranted to evidence the redundancy of lipid internalization/receptor function, their co‐adaptors and monocyte/macrophage polarization characteristics in FH patient's cells and their contribution to atherosclerotic vascular remodelling and progression.

## Author contributions

RE performed the research and statistical analyses of the study, interpreted the results and wrote the manuscript. TP assisted in designing the research study, performed statistical analysis, interpreted the results, wrote the manuscript and obtained funding. MB performed the LRP5 silencing experiments and the mice model studies and revised the manuscript. RS contributed to patient selection, statistical analysis and revised the manuscript. RA contributed to the research study and revised the manuscript. PM obtained the biological samples and clinical data of the FH and non‐FH patient from the SAFEHEART cohort and revised the manuscript. LB designed the research study, interpreted the results, wrote the manuscript and obtained funding.

## Conflicts of interest

The authors confirm that there is no conflict of interest.

## Supporting information


**Fig. S1** Schematic diagram representing the study design with 205 individuals of the SAFEHEART Cohort.
**Fig. S2** LRP5 expression in PBL from FH‐AT patients.
**Fig. S3** mRNA expression levels of LRPs and scavenger receptors in macrophages of FH patients according their genetically characterized LDLR mutation.
**Fig. S4** *Cd163* expression is up‐regulated in PBLs of *Lrp5* ^*−/−*^ mice.
**Table S1** Clinical characteristics of control LLT^−^, FH‐LLT^+^ and FH‐LLT^−^.
**Table S2** Clinical characteristics of sc‐HC and FH‐AT donors for studies on peripheral blood leucocytes (PBL).
**Table S3** Clinical characteristics of control and FH donors for studies on monocyte differentiation to macrophage.
**Table S4** FH genotypes according to location of LDLR mutation.
