# Supplementary material for: Macrophages of genetically characterized familial hypercholesterolaemia patients show up‐regulation of LDL‐receptor‐related proteins
Source: J Cell Mol Med. 2016 Sep 29;21(3):487–99. doi: 10.1111/jcmm.12993 (PMC5323824; doi:10.1111/jcmm.12993)
Supplement: Supplementary file 1 — Fig. S1 Schematic diagram representing the study design with 205 individuals of the SAFEHEART Cohort. Fig. S2 LRP5 expression in PBL from FH‐AT patients. Fig. S3 mRNA expression levels of LRPs and scavenger receptors in macrophages of FH patients according their genetically characterized LDLR mutation. Fig. S4 Cd163 expression is up‐regulated in PBLs of Lrp5 −/− mice. Table S1 Clinical characteristics of control LLT−, FH‐LLT+ and FH‐LLT−. Table S2 Clinical characteristics of sc‐HC and FH‐AT donors for studies on peripheral blood leucocytes (PBL). Table S3 Clinical characteristics of control and FH donors for studies on monocyte differentiation to macrophage. Table S4 FH genotypes according to location of LDLR mutation. [file JCMM-21-487-s001.pdf]

MACROPHAGES OF GENETICALLY CHARACTERIZED FAMILIAL HYPERCHOLESTEROLEMIA PATIENTS SHOW UPREGULATION OF LDL-RECEPTOR RELATED PROTEINS

Rafael Escate [a] , Teresa Padro [a], Maria Borrell-Pages [a], Rosa Suades [a], Rosa Aledo [a], Pedro Mata [c], Lina Badimon [a, b, *]

[a] Cardiovascular Research Center (CSIC-ICCC), IIB-Sant Pau, Barcelona, Spain;

[b] Cardiovascular Research Chair, UAB, Barcelona, Spain

[c] Foundation Jimenez Diaz, Madrid, Spain

***Correspondence to:** Prof. Lina Badimon
Cardiovascular Research Center
Hospital de la Santa Creu I Sant Pau,
IIB-Sant Pau, Barcelona, Spain.
Sant Antoni Maria Claret 167, 08025 Barcelona, Spain.
Phone: +34.935565880.
Fax: +34.935565559.
E-mail: lbadimon@csic-iccc.org

Supporting Information: 4 Supplementary Figures and 4 Supplementary Tables

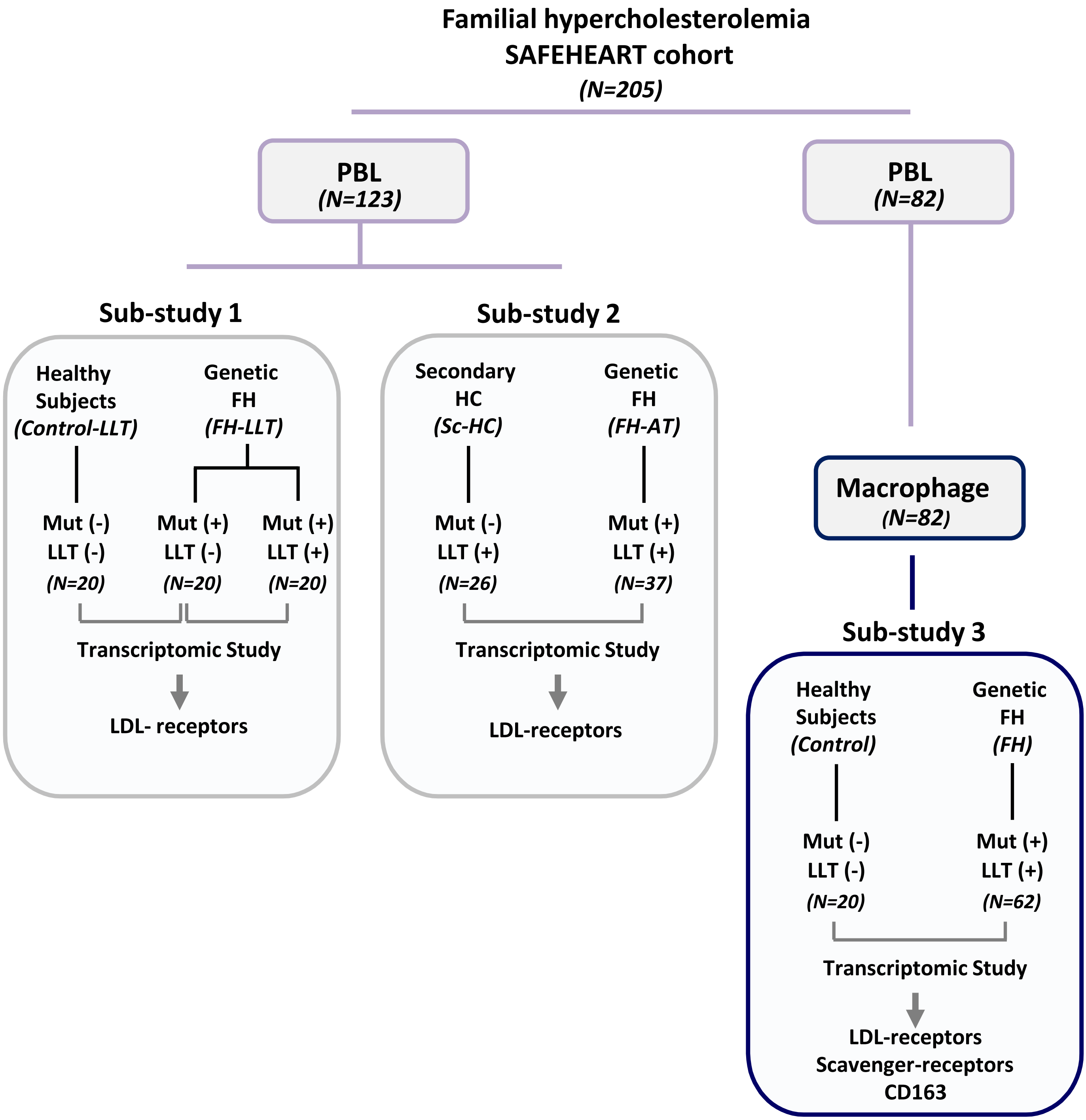

Supplementary Figure 1. Schematic diagram representing the study design with 205 subjects of the SAFEHEART Cohort

FH: Familial hypercholesterolemia; HC: hypercholesterolemia; Mut (+): With LDLR mutation, Mut (-): Without LDLR mutation; LLT (+): with lipid-lowering treatment; LLT (-): without lipid-lowering treatment. Secondary HC is shown in manuscript as sc-HC.

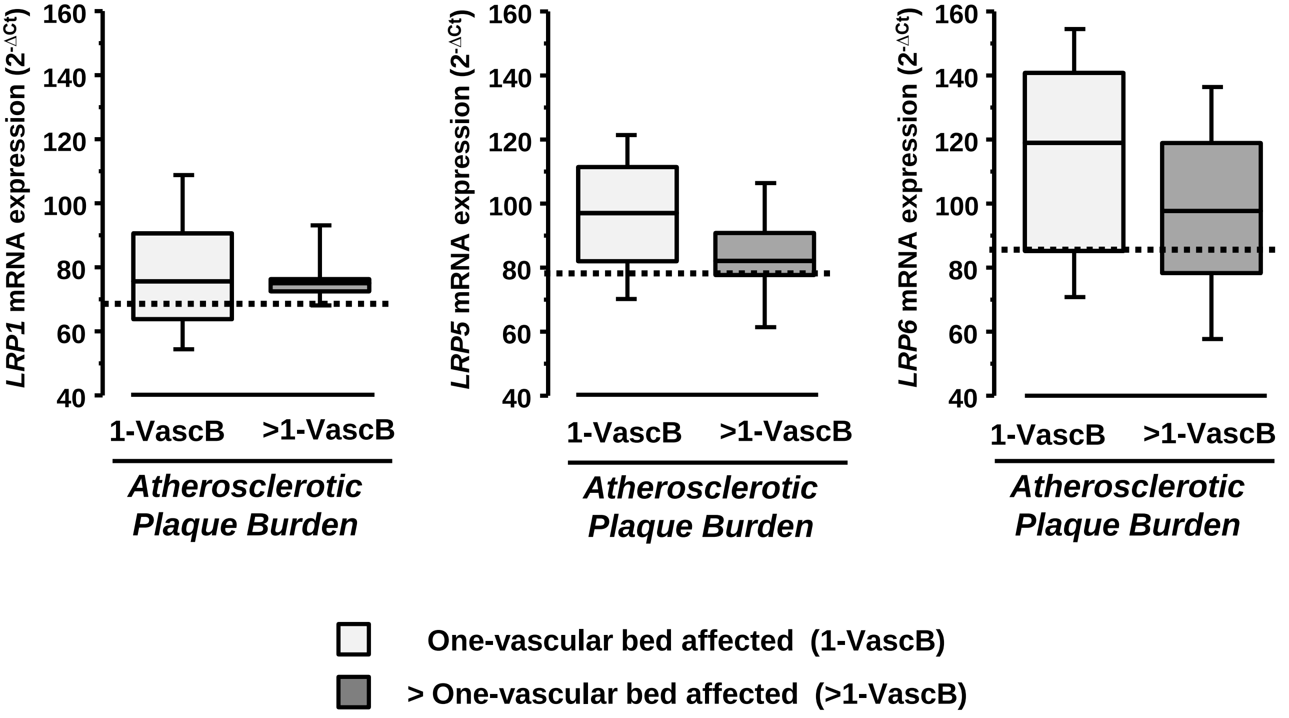

Supplementary Figure 2. LRP5 expression in PBL from FH-AT patients

Box and whisker plot of LRP1, LRP5 and LRP6 mRNA expression associated to atherosclerotic plaque burden in FH-patients. Horizontal doted lines indicate median levels of mRNA expression ($2^{-\Delta Ct}$) of the control sc-HC group for LRP1 (68), LRP5 (79), and LRP6 (84). Thirty FH-AT patients only had one vascular bed affected (aortic or carotid) and 7 patients had atherosclerotic burden in the aortic and carotid beds. Values are give as median and interquatile range. Differences between groups are not statistically significant (U-Mann Whitney).

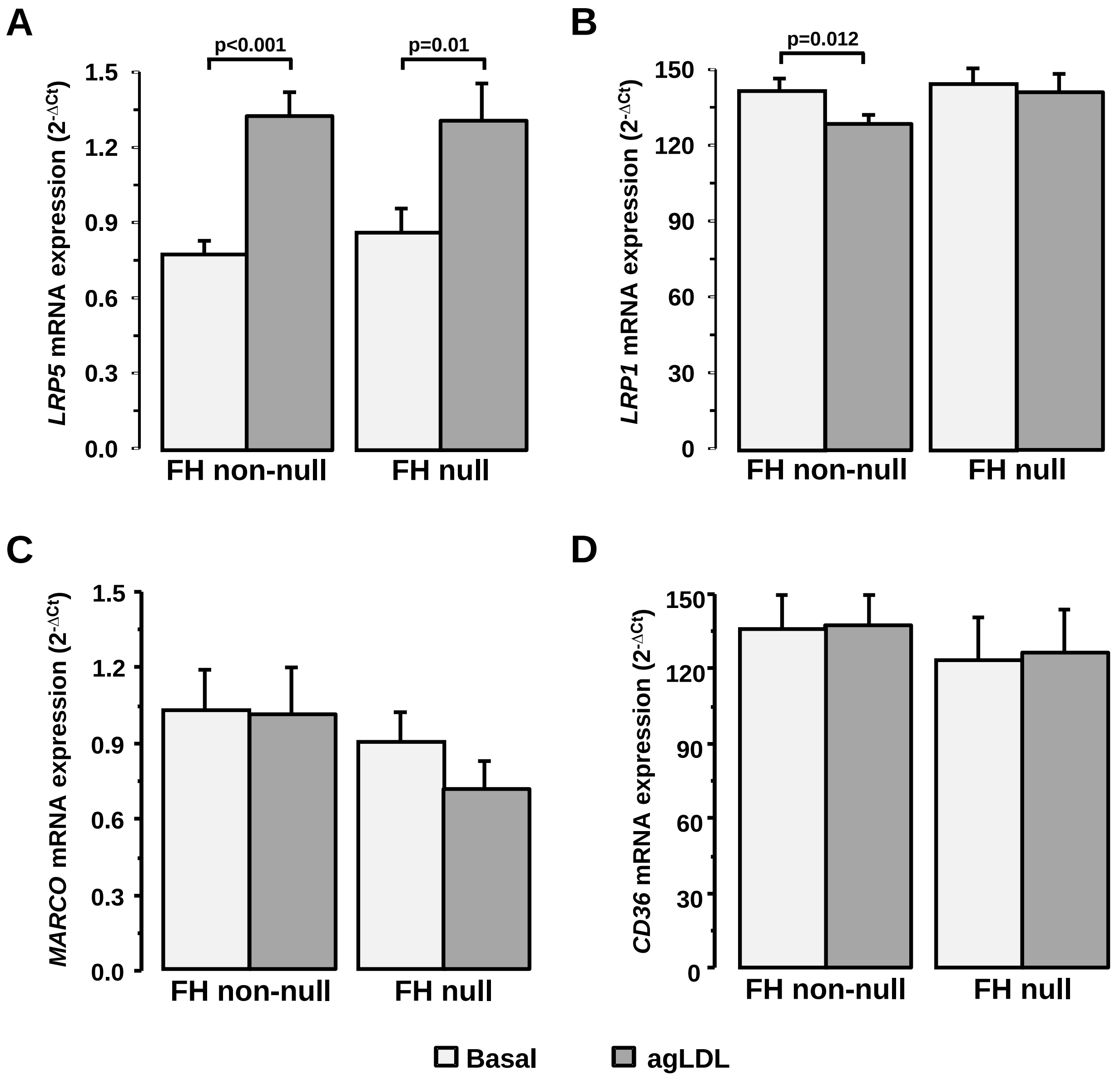

Supplementary Figure 3. mRNA expression levels of LRPs and scavenger receptors in macrophages of FH-patients according their genetically characterized ldlr-mutation. Macrophage from LDLR-non-null (FH non-null, N=40) and LDLR-null (FH null, N=22) FH-patients were incubated with 100µg/mL agLDL for 24 hours. mRNA expression was quantified by real time PCR in sample duplicates. Bars represent mean±SEM. Only p-values <0.05 are shown.

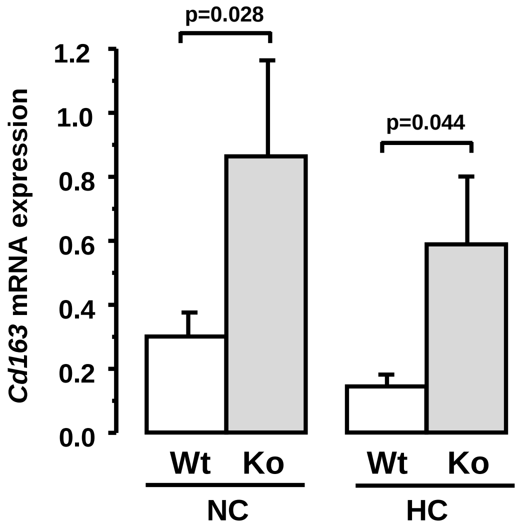

Supplementary Figure 4. ***Cd163*** **expression is upregulated in PBLs of** ***Lrp5*** $^{-/-}$ **mice.** Differential expression of *Cd163* in PBLs of wild type (Wt) and *Lrp5* $^{-/-}$ (Ko) mice fed hypercholesterolemic (HC) and normocholesterolemic (NC) diet. Real-time PCR in duplicates and normalized to 18S (N= 7 mice/group). Bars represent mean±SEM. Only p-values <0.05 are shown.

Supplementary Table 1. Clinical characteristics of Control-LLT$^-$, FH-LLT$^+$ and FH-LLT$^-$ donors for studies on peripheral blood leukocytes (PBL).

	Control-LLT$^-$ *n=20*	FH-LLT$^+$ *n* =20	FH-LLT$^-$ *n* =20	Statistics
Age (years, mean ± SEM)	35 ± 2	37 ± 2	34 ± 1	p=0.501
Male/Female, *n*	10/10	10/10	10/10	-
Body mass index (Kg/m^2, mean ± SEM)	23.3 ± 0.9	24.7 ± 1.1	24.6 ± 0.9	p=0.646
Waist to height ratio (mean ± SEM)	0.5 ± 0.3	0.48 ± 0.2	0.47 ± 0.1	p=0.973
**Risk Factors, *n* (%)**				
Diabetes mellitus	0 (0%)	0 (0%)	0 (0%)	-
Systemic hypertension	0 (0%)	0 (0%)	0 (0%)	-
Obesity (BMI>30)	1 (5%)	3 (30%)	2 (10%)	p=0.574
Current tobacco consumption	5 (25%)	9 (45%)	9 (45%)	p=0.324
**Lipid-lowering therapy, *n* (%)**				
Statins	0 (0%)	20 (100%)	0 (0%)	p<0.001
Ezetimibe	0 (0%)	0 (0%)	0 (0%)	
Statin treatment time (years, mean ± SEM)	0	15 ± 1	0	p<0.001
**Clinical data (mean ± SEM)**				
Total cholesterol (mg/dL)	167.9 ± 4.2	296.9 ± 7.5[a]	302.9 ± 6.4[a]	p<0.001
LDL-cholesterol (mg/dL)	92.7 ± 3.4	222.7 ± 6[a]	231.3 ± 6.9[a]	p<0.001
HDL-cholesterol (mg/dL)	60.1 ± 4.3	55.9 ± 3.4	53.6 ± 2.4[a]	p=0.749
Non-HDL-cholesterol (mg/dL)	107.8 ± 3.3	240.9 ± 7.1[a]	249.3 ± 6.4[a]	p<0.001
TC/HDL-cholesterol ratio	2.8 ± 0.1	5.6 ± 0.3[a]	5.9 ± 0.3[a]	p<0.001
Triglyceride (mg/dL)	75.1 ± 8.1	90.5 ± 10.1	87.2 ± 8.6	p=0.251
Hight-sensitive CRP (mg/dL)	1.7 ± 0.4	2.8 ± 0.9	1.6 ± 0.3	p=0.701
Fasting plasma glucose (mg/dL)	82.4 ± 2.4	77.9 ± 2.1	79.1 ± 1.9	p=0.385

Control-LLT$^-$ group: healthy subjects without lipid-lowering therapy; FH-LLT$^+$ and FH-LLT$^-$ group: FH-patients with or without LLT; BMI: body mass index; LDL: low-density lipoprotein; HDL: high-density lipoprotein; TC: total cholesterol and CRP: C-reactive protein. [a] p-values respect to Control-LLT$^-$ group.

Supplementary Table 2. Clinical characteristics of sc-HC and FH-AT donors for studies on peripheral blood leukocytes (PBL).

	**sc-HC** ***n*** **=26**	**FH-AT** ***n*** **=37**	**Statistics**
Age (years, mean ± SEM)	56 ± 3	44 ± 2	p=0.001
Male/Female, *n*	10/16	19/18	p=0.312
Body mass index (Kg/m^2, mean ± SEM)	28.2 ± 1.3	26 ± 0.7	p=0.138
Waist to height ratio (mean ± SEM)	0.57 ± 0.2	0.50 ± 0.01	p=0.004
**Risk Factors, *n* (%)**			
Diabetes mellitus	2 (8%)	0 (0%)	p=0.086
Systemic hypertension	5 (19%)	4 (11%)	p=0.347
Obesity (BMI>30)	10 (38%)	3 (8%)	p=0.003
Current tobacco consumption	7 (27%)	13 (35%)	p=0.491
**Lipid-lowering therapy, *n* (%)**			
Statins	26 (100%)	37 (100%)	-
Ezetimibe	2 (8%)	13 (35%)	p=0.012
Statin treatment time (years, mean ± SEM)	12 ± 1	11 ± 1	p=0.626
**Clinical data (mean ± SEM)**			
Total cholesterol (mg/dL)	212.5 ± 7.5	232.9 ± 12.6	p=0.418
LDL-cholesterol (mg/dL)	136.2 ± 6.8	160.1 ± 10.7	p=0.133
HDL-cholesterol (mg/dL)	56.4 ± 2.8	49.6 ± 2.1	p=0.077
Non-HDL-cholesterol (mg/dL)	156 ± 7.2	182 ± 12.2	p=0.176
TC/HDL-cholesterol ratio	3.9 ± 0.2	4.9 ± 0.3	p=0.036
Triglyceride (mg/dL)	99.2 ± 9.5	39 ± 6.4	p<0.001
Hight-sensitive CRP (mg/dL)	3.1 ± 0.9	2.9 ± 0.6	p=0.469
Fasting plasma glucose (mg/dL)	92.3 ± 3.8	87.4 ± 3.2	p=0.459

Sc-HC group: Subjects with secondary hypercholesterolemia and FH-AT group: FH-patients with atherosclerotic lesion analyzed with magnetic resonance image; BMI: body mass index; LDL: low-density lipoprotein; HDL: high-density lipoprotein; TC: total cholesterol and CRP: C-reactive protein.

Supplementary Table 3. Clinical characteristics of control and FH donors for studies on monocyte differentiation to macrophage.

	**Control** *n* **=20**	**FH** *n* **=62**	**Statistics**
Age (years, mean ± SEM)	47 ± 3	46 ± 2	p=0.888
Male/Female, *n*	11/9	31/31	p=0.697
Body mass index (Kg/m^2, mean ± SEM)	25.6 ± 1.1	26.9 ± 0.7	p=0.483
Waist to height ratio (mean ± SEM)	0.53 ± 0.02	0.56 ± 0.01	p=0.329
**Risk Factors, *n* (%)**			
Diabetes mellitus	0 (0%)	3 (4.8%)	p=0.316
Systemic hypertension	1 (5%)	6 (9.7%)	p=0.515
Obesity (BMI>30)	4 (20%)	13 (21%)	p=0.325
Current tobacco consumption	8 (40%)	19 (30.6%)	p=0.438
**Lipid-lowering therapy, *n* (%)**			
Statins	9 (45%)	62 (100%)	p<0.001
Ezetimibe	0 (0%)	2 (1.2%)	p=0.416
Statin treatment time (years, mean ± SEM)	3 ± 1	15 ± 1	p<0.001
**Clinical data (mean ± SEM)**			
Total cholesterol (mg/dL)	188.3 ± 4.6	243.6 ± 7.4	p<0.001
LDL-cholesterol (mg/dL)	117.6 ± 4	176.7 ± 6.8	p<0.001
HDL-cholesterol (mg/dL)	47.2 ± 2.6	49 ± 1.3	p=0.496
Non-HDL-cholesterol (mg/dL)	141.1 ± 4	195 ± 7.4	p<0.001
TC/HDL-cholesterol ratio	4.2 ± 0.2	5.2 ± 0.2	p=0.003
Triglyceride (mg/dL)	135.9 ± 23.6	89.3 ± 6.5	p=0.063
Hight-sensitive CRP (mg/dL)	2.1 ± 0.4	1.5 ± 0.2	p=0.457
Fasting plasma glucose (mg/dL)	80.2 ± 2.4	81.4 ± 1.7	p=0.756

Control group (C-MAC): healthy subjects with or without lipid-lowering therapy (LLT); FH group (FH-MAC): FH patients with LLT; BMI: body mass index; LDL: low-density lipoprotein; HDL: high-density lipoprotein; TC: total cholesterol and CRP: C-reactive protein.

Supplementary Table 4. FH genotypes according to location of LDLR mutation

FH group	Location	Predicted effect	N=62
null	Ex 15	Shift reading frame	10
null	Ex 7		
null	Ex 6		
null	Ex 4	Stop codon	12
null	Ex 9		
null	Ex 2		
non-null	Prom	Regulation of expression	4
non-null			
non-null	Ex 17	Change amino acid	15
non-null	Ex 3		
non-null	Ex 4		
non-null	Ex 7		
non-null	Ex 14		
non-null	Ex 8		
non-null	Int 3	Splicing	9
non-null	Int 16		
non-null	Int 9		
non-null	Int 12		
non-null	Ex 3 + Int 3	Others	12
non-null	Ex 13		
non-null	Ex 11 + Ex 17		
non-null	Ex 16		

Stop codon and shift reading frame mutations were classified as FH null (N=22) while the other mutations were classified as FH non-null (N=40). Ex: exon; Int: intron; Prom: promoter; N: number of patients for each predicted effect of LDLR mutation.
